# Association between thigh muscle strength four years after partial meniscectomy and radiographic features of osteoarthritis 11 years later

**DOI:** 10.1186/s12891-019-2875-7

**Published:** 2019-11-03

**Authors:** Ylva B. Ericsson, Ewa M. Roos, Henrik Owman, Leif E. Dahlberg

**Affiliations:** 1 Department of Clinical Sciences Malmö, Lund University, Lund, Sweden; 2 Department of Orthopaedics, Skane University Hospital, SE-205 02 Malmö, Sweden; 3 Research Unit for Musculoskeletal Function and Physiotherapy, Department of Sports Science and Clinical Biomechanics, University of Southern Denmark, Odense, Denmark; 4 Department of Clinical Sciences Lund, Orthopaedics, Lund University, Skane University Hospital, Lund, Sweden

**Keywords:** Meniscectomy, Knee osteoarthritis, Joint space narrowing, Osteophytes, Muscle strength

## Abstract

**Background:**

Meniscus injury and meniscectomy both entail increased risk of knee osteoarthritis (OA). Thigh muscle weakness is a suggested mediator of OA but there is little evidence of its importance for knee OA development after meniscectomy. This study aimed to examine the association between thigh muscle strength after partial meniscectomy in middle-aged subjects with a non-traumatic meniscal tear and later radiographic knee OA changes.

**Methods:**

Thirty-four out of 45 participants in an exercise-trial underwent testing for isokinetic thigh muscle strength 4 years after arthroscopic partial meniscectomy and had radiographic examination 11 years later (15 years post-surgery, mean age at follow-up of 57 years (range 50–61)). Outcomes were grade of joint space narrowing and osteophyte score in the medial tibiofemoral compartment of the operated knee and the contralateral knee. We tested the association between muscle strength at baseline and the radiographic outcomes at follow-up using logistic regression analyses adjusted for sex and overweight.

**Results:**

At follow-up, 33/34 subjects had joint space narrowing and 27/34 subjects had osteophytes in the operated knee, in the contralateral knee joint space narrowing was found in 23 subjects. In the operated knee baseline knee extensor and flexor strength were negatively associated with grade of joint space narrowing at follow-up (OR 0.972 and 0.956, *p* = 0.028 and 0.026, respectively) and also with osteophyte score (OR 0.968 and 0.931, *p* = 0.017 and 0.011, respectively). In the contralateral knee longitudinal associations between strength and radiographic OA features were similar, OR 0.949–0.972, *p* < 0.05.

**Conclusion:**

The finding that stronger thigh muscles 4 years after meniscectomy were associated with less severe osteoarthritic changes in the medial tibiofemoral compartment of both the operated and contralateral knee 11 years later, may suggest that strong thigh muscles can help to preserve joint integrity in middle-aged subjects at risk of knee OA.

## Background

The menisci have important shock absorbing and load transmitting functions and contribute also to joint stability [1–3]. In middle-aged or elderly subjects it is common that meniscal tears occurs after a minor trauma as the meniscus is undergoing degenerative processes [4, 5]. Meniscal tear is a well-known risk factor for knee osteoarthritis (OA) [4], and according to previous studies about half of meniscus surgery patients develop radiographic OA 10–20 years after meniscectomy [5–8].

In the general population thigh muscle weakness has been suggested to be an independent risk factor for developing symptomatic or radiographic knee OA in both men and women [9, 10], and quadriceps weakness has been reported up to 5 years after arthroscopic partial meniscectomy (APM) [11–14]. However, the independent effect of thigh muscle weakness on knee OA after meniscectomy has not been elucidated. Weak thigh muscles may expose the articular cartilage to harmful loading since dynamic stability as well as shock absorbing function of the muscles are impaired [15, 16]. After meniscal injury and surgery, joint biomechanics may be altered which puts additional stress on the cartilage [17, 18]. Identifying the relative importance of modifiable factors associated with knee OA development after meniscectomy could help improve strategies for prevention of knee OA.

In a middle-aged post meniscectomy group which we examined 4 years after surgery, we found moderate strength deficits, pain and functional limitations [12], a positive cross-sectional correlation between thigh muscle strength and knee cartilage quality [19] and also improvement in cartilage quality after a 4 month exercise intervention [20].

With the hypothesis that strong thigh muscles may protect against knee OA development in subjects who have had a meniscal injury and a subsequent meniscectomy, we aimed to examine the association between thigh muscle strength at 4 yrs after APM due to a non-traumatic tear and radiographic osteoarthritis features in the operated knee 11 years later. Secondary aims were to examine the longitudinal association between muscle strength and OA changes in the contralateral knee, and to examine the cross-sectional association between muscle strength and OA features at follow-up.

## Methods

### Subjects at baseline examination in 2001

Forty-five middle-aged subjects (16 women) who participated in an exercise trial post-meniscectomy [20], and underwent testing for muscle strength in 2001, 4 yrs after surgery, were invited to participate in this follow-up conducted 15 years after surgery, i.e.11 years after muscle strength was examined. All subjects had had an APM due to a non-traumatic tear at the Orthopedic Department at Skane University Hospital, at the ages of 35–45, during the years 1996–2000. Surgery was performed by several surgeons. All meniscus tears were reported to be of degenerative type and only the damaged parts of the torn menisci were excised. In the arthroscopy reports the cartilage of the medial femoral condyle was described as normal in 21 patients, with shallow lesions in 20 cases and with localized full-thickness lesions in four cases. In all patients, the cartilage in the lateral compartment was described as normal. Thus no cartilage changes were defined as deep clefts or visible bone.

Subjects received a written invitation together with information about the follow-up study, and thereafter, if needed, two reminder letters.

### Subjects at the follow-up examination in 2012–2013

Thirty-four subjects (11 women) with mean age of 57 (range 50–61) years at follow-up accepted to participate in the follow-up assessment in 2012–2013. All subjects signed an informed consent form. Eleven subjects did not participate as they were either not interested, had lack of time, or had relocated. These 11 subjects lost to follow up did not differ significantly from the study group with respect to age, gender, BMI or muscle strength at baseline (*p* = 0.17–0.78).

**Fig. 1:**
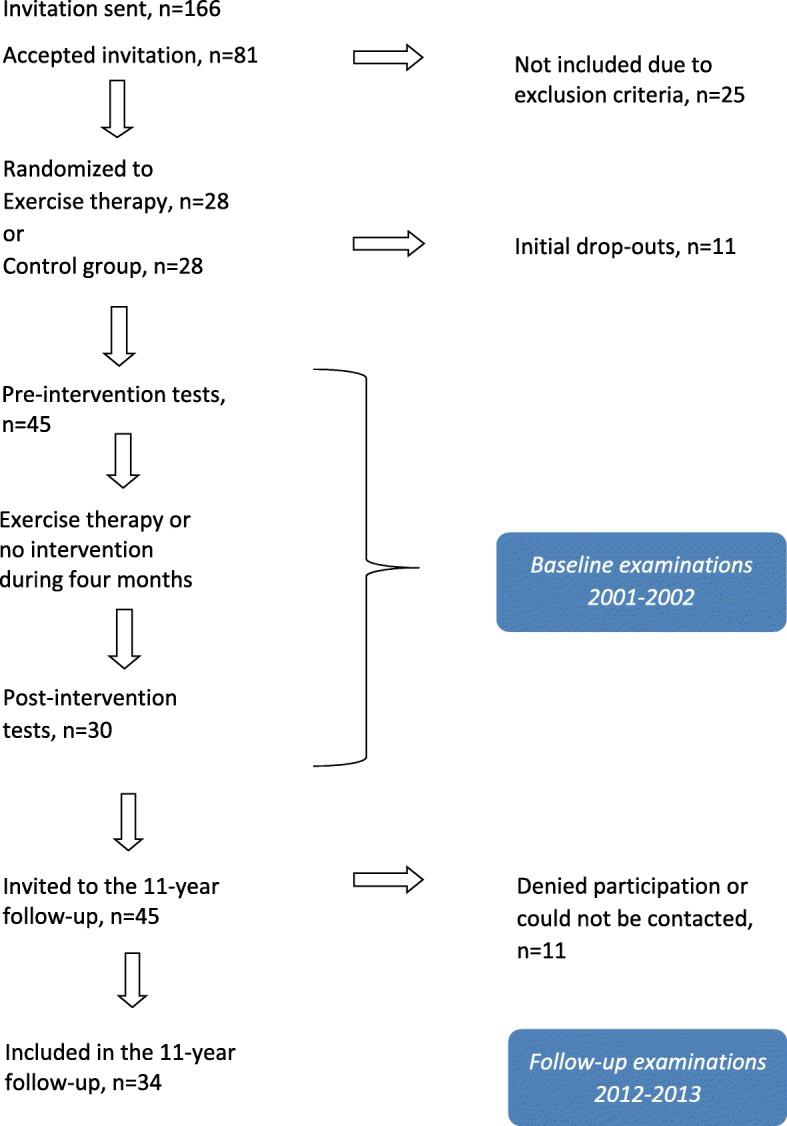
Flowchart of the patients in the study

The flow of patients is illustrated in Fig. 1. In this report we refer to the muscle strength examinations of the patient cohort performed pre- and post-intervention in 2001–2002 as *baseline*, and to the follow-up including radiographs in 2012–2013 as *follow-up*. At the follow-up visit patients were assessed at the hospital for 2 h.

### The original study

Purpose of the RCT was to evaluate the effects of moderate exercise on glycosaminoglycan (GAG) content in knee cartilage in subjects at high risk of knee OA [20]. Forty-five subjects who underwent partial medial meniscectomy 3–5 years previously and agreed to participate in the study were randomized to either supervised group-based exercise therapy or no intervention during 4 months. The exercise therapy which was individualized to each subject aimed to improve muscle strength and neuromuscular control in the lower extremities. Subjects were expected to attend exercise classes lasting 1 h 3 times a week. The primary endpoint was change in cartilage quality measured as cartilage GAG content estimated with delayed gadolinium-enhanced MRI of cartilage (dGEMRIC). Subjects were assessed before and after the 4 month intervention regarding cartilage quality and muscle strength and asked to complete questionnaires concerning knee symptoms and function and physical activity level [20].

### Muscle strength at baseline

For the purpose of this longitudinal analysis, we have used the highest muscle strength value obtained from either before or after the four-month intervention comparing neuromuscular exercise to no treatment performed in 2001. With slight variations in number of subjects for extensor and flexor strength, and the operated and contralateral legs respectively, the pre-intervention values were used for about half of the subjects and the post-intervention values for the other half of the subjects.

### Follow-up radiographs

Radiographs were obtained in 2012–2013, 11 years after baseline tests and equivalent to 12–16 years after meniscectomy.

#### Assessments at baseline and follow-up

### Isokinetic muscle strength testing

Strength tests for knee extensors and knee flexors of both knees was conducted with Biodex System 3 Pro [21]. The test was performed at an angular velocity of 60 degrees/second in the range of 10 to 95 degrees of flexion. Muscle strength was measured as concentric peak torque (PT) in Newton meters (Nm), the value obtained was normalized to body weight and expressed as Nm/kg × 100.

### Body height and body weight measurement for calculation of BMI

BMI was calculated by obtaining height and weight using a wall-mounted ruler and a calibrated scale. The standardized formula for BMI (kg/m^2^) was employed.

#### Additional assessment at follow-up

### Radiographic examination

While the patient was standing with semi-flexed knees posterior-anterior radiographs were obtained from both knee joints. Radiographs were read by two observers separately, blinded to clinical status and initial randomization group. In cases of discrepancy, the radiographs were re-read and discussed until consensus was reached. The OA features were graded as follows:

Joint space narrowing (JSN) and femoral and tibial osteophytes were individually graded on frontal images using a 4-point scale (0–3, 0 = no evidence of JSN or bony change) according to the Osteoarthritis Research Society International Atlas [22]. Medial and lateral osteophyte scores were calculated for each knee, by summing the femoral and the tibial osteophyte grades in the medial and lateral compartments, respectively (i.e., yielding two scores ranging from 0 to 6). In the present study we were especially interested in OA features in the *medial* TF compartment as patients had undergone *medial meniscectomy* and hence we used the medial JSN grade and medial osteophyte score as dependents in the regression analyses.

Radiographic TF knee OA was considered present if any of the following criteria was fulfilled in either of the two TF compartments [23].

- JSN grade ≥ 2
- osteophyte score ≥ 2
- JSN grade 1 in combination with osteophyte grade 1 in the same compartment

This definition of TF OA approximates grade 2 knee OA or worse based on the Kellgren and Lawrence scale [24].

#### Statistics

We tested the association between baseline muscle strength (best value obtained at pre- or post-exercise intervention) and radiographic OA features (in the medial compartment of the TF joint) at the 11-year follow-up using logistic regression analyses with overweight (BMI ≥25) and sex as control variables. Dependents were higher /lower JSN grade (≥2/< 2) and higher/lower osteophyte score (≥2/ < 2). Knee extensor strength and knee flexor strength were studied in separate models to avoid multicollinearity. Results from both unadjusted and adjusted analyses are presented. Differences between baseline and follow-up in muscle strength and BMI were determined with the Wilcoxon signed rank test. To compare radiographic outcomes in men and women we used the Chi-Square test. Two-tailed *P*-values < 0.05 were considered statistically significant.

## Results

### Results of the thirty-four subjects that participated in the follow-up

The characteristics of the patients who participated in the follow-up are shown in Table 1 together with their clinical results at baseline and follow up assessments.

**Table 1:** Characteristics and clinical outcome of the study group (*N* = 34)

	Baseline	Follow-up	P*
Sex, n (%) female	11 (32)		
Age, mean ± SD, years	45.6 ± 3.2	56.9 ± 2.9	
Follow-up time after surgery, mean ± SD, years	3.7 ± 1.1	14.9 ± 1.1	
Physical activity level (low/ high), n (%)	11 (32)/23 (68)	10 (30)/23 (70)	
Body Mass Index, mean ± SD years	26.0 ± 3.0	26.6 ± 3.6	0.045
Overweight (BMI ≥ 25), n (%)	23 (68)	22 (65)	
Muscle strength, md (IQR), Nm/kg			
Knee extensor strength operated leg	205.5 (52)	184 (63)	< 0.001
Knee extensor strength contralateral leg	222.5 (71)	169.8 (63)	< 0.001
Knee flexor strength operated leg	117 (38)	94.7 (44)	< 0.001
Knee flexor strength contralateral leg	110 (50)	93.8 (32)	< 0.001

Knee extensor and knee flexor strength was measured as body weight adjusted peak torque; Physical activity levels were self-reported. * By Wilcoxons signed rank test

### Thigh muscle strength and BMI at baseline and follow-up

At baseline examination, mean age was 45.6 years and mean BMI 26.0 and 23 subjects were overweight according to the WHO definition of BMI ≥ 25. On the individual level most subjects had strength deficits in the operated leg compared to the other leg, with a median difference of 8% (range − 31%- + 11%) for knee extensors (*p* < 0.001), and median difference of 6% (range − 37 - + 44%) for knee flexors (*p* = 0.169). On group level there was a significant difference between operated legs and contralateral legs regarding knee extensor strength (*p* ≤ 0.001) but not knee flexor strength (p = 0.169). At follow-up, mean BMI had increased to 26.6 (*p* = 0.045), 22 subjects were overweight, and knee extensor and knee flexor strength of the operated leg had decreased with a median of 10 and 19%, respectively (*p* < 0.001), Table 1 and were similar to the strength of the contralateral leg (*p*> 0.30).

### Radiographic features of knee OA in the TF joint at follow-up

#### JSN

**Fig. 2:**
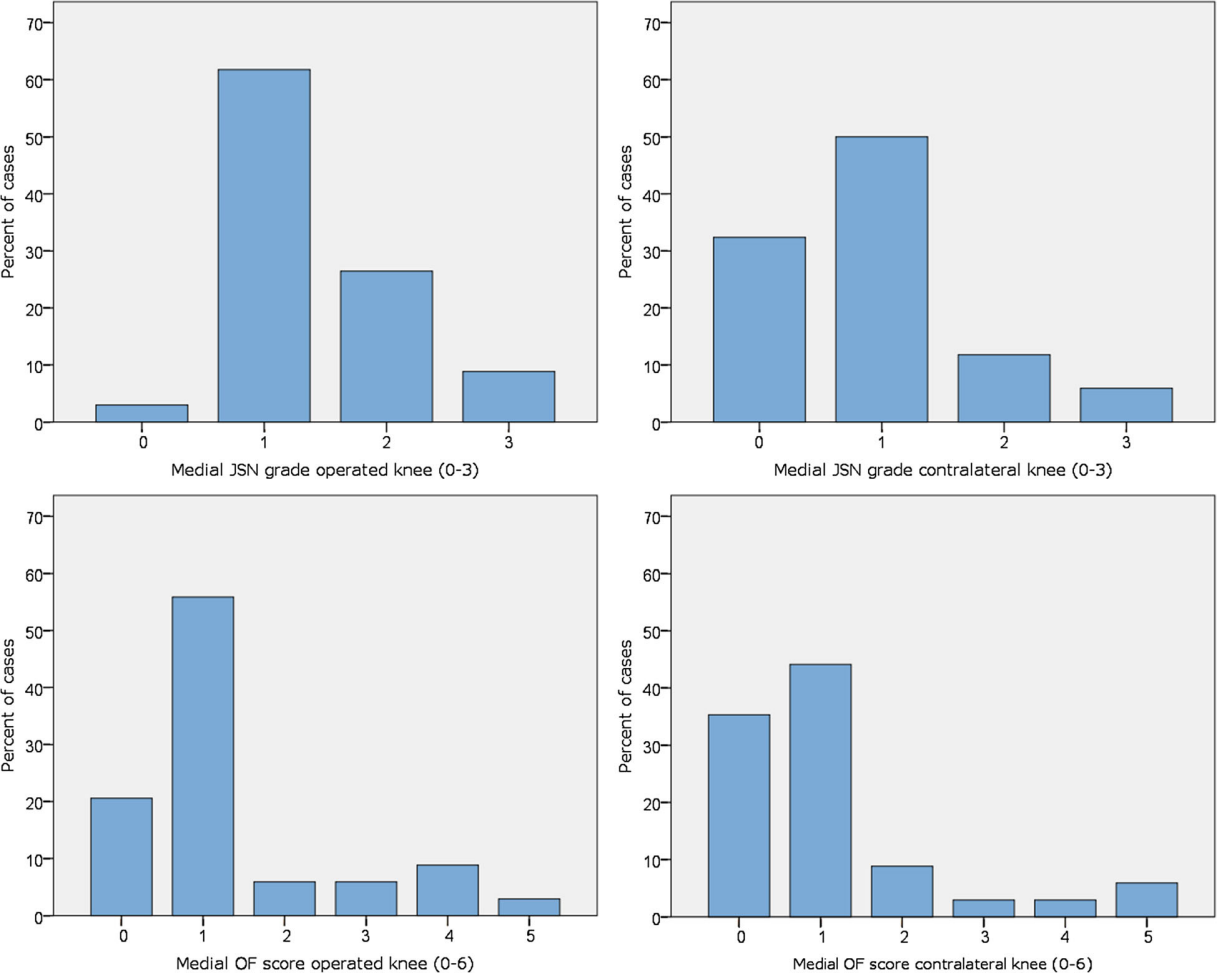
Distribution of radiographic OA features in operated and contralateral knees at follow-up. *JSN grade refers to grade of Joint Space Narrowing in the medial tibiofemoral compartment; OF score refers to osteophyte score in the same compartment

All 34 subjects had JSN in the TF joint of the operated knee, varying between grade 1 and grade 3 (Fig. 2). Thirty-three subjects had JSN in the medial compartment, one patient in the lateral compartment, and two patients had JSN in both medial and lateral compartments of the operated knee. In the contralateral knee, 23 subjects had JSN of varying grade in the medial compartment (Fig. 2). Men and women had the same distribution of higher/lower medial JSN grade in the operated knee (*p* = 0.10) and in the contralateral knee (*p* = 0.31).

#### Osteophytes

In the medial compartment of the operated knee 27 out of 34 patients had osteophytes of varying numbers and grades and the corresponding number for the contralateral knee was 22 patients (Fig. 2). Lateral compartment osteophytes were found in the operated knee in 12 patients and in the contralateral knee in two patients. Men and women had the same distribution of higher/lower medial osteophyte score in the operated knee (*p* = 0.61) and in the contralateral knee (*p* = 0.15).

### Radiographic knee OA according to the OARSI atlas criteria

Twenty-seven out of 34 subjects were classified as having OA in the TF joint of the operated knee. Sixteen subjects had TF OA in the contralateral knee and 13 subjects had TF OA in both knees. Men and women had the same OA prevalence in the operated knee (*p* = 0.51) and in the contralateral knee (*p* = 0.18) but more women than men had bilateral knee OA (*p* = 0.035).

### Longitudinal associations between thigh muscle strength and radiographic knee OA features

#### Operated knee

##### JSN in the medial compartment of the TF joint

**Fig. 3:**
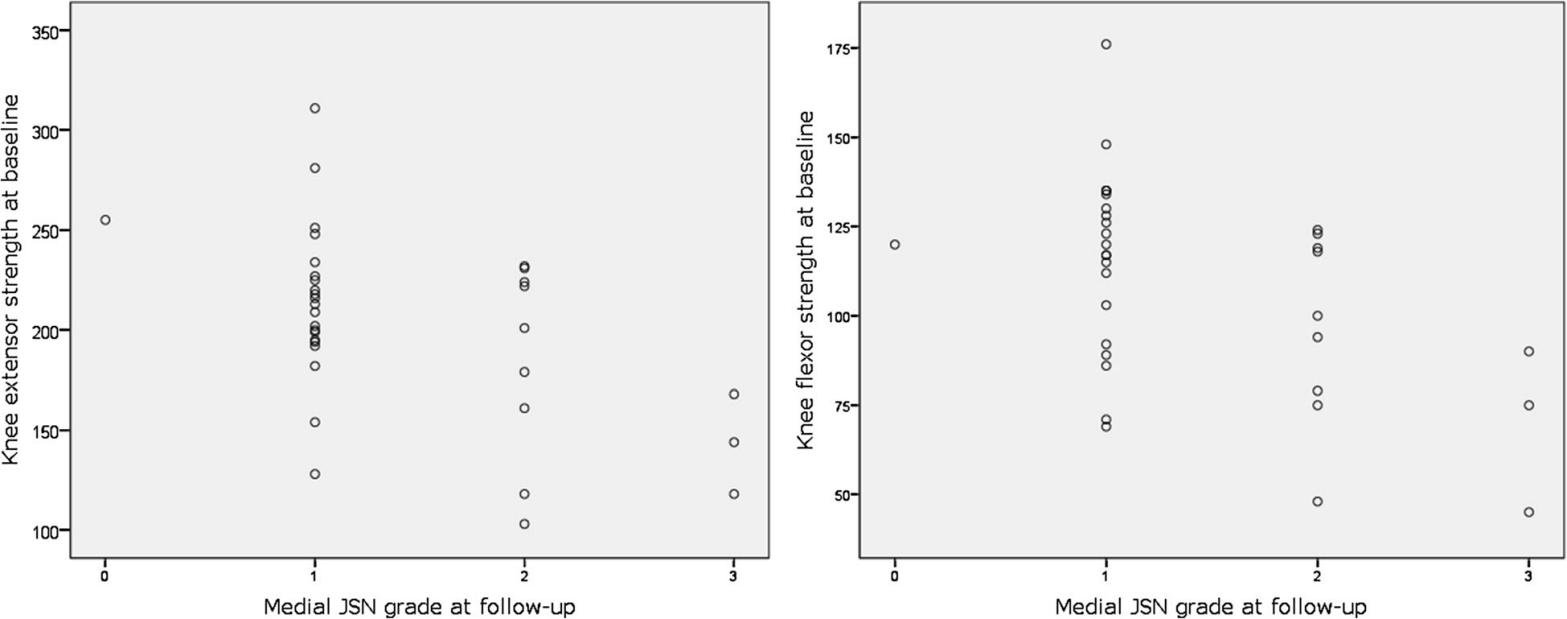
Relationship between baseline thigh muscle strength and JSN grade in the operated knee at follow-up. *Medial JSN grade refers to grade of Joint Space Narrowing in the medial tibiofemoral compartment of the operated knee

On the operated side we found a negative association between baseline knee extensor and knee flexor strength and JSN grade at follow-up (Fig. 3), both in the unadjusted models (OR 0.977 and 0.961, *p* = 0.022 and 0.017, respectively (Table 2), and in the adjusted models with sex and overweight as control variables, OR 0.972 and 0.956, *p* = 0.028 and 0.026, respectively (Table 2).

**Table 2:** Association between baseline thigh muscle strength and radiographic knee OA features at follow-up*

	Unadjusted			Adjusted		
Predictor	OR	95% CI	*P*	OR	95% CI	*P*
Muscle strength, operated leg	JSN grade† operated knee					
Knee extensors	0.977	0.957–0.997	0.022	0.972	0.947–0.997	0.028
Knee flexors	0.961	0.930–0.993	0.017	0.956	0.919–0.994	0.026
	Osteophyte score†† operated knee					
Knee extensors	0.966	0.942–0.991	0.008	0.968	0.943–0.994	0.017
Knee flexors	0.936	0.893–0.981	0.006	0.931	0.881–0.984	0.011
Muscle strength, contralateral leg	JSN grade† contralateral knee					
Knee extensors	0.961	0.930–0.993	0.017	0.968	0.940–0.996	0.027
Knee flexors	0.967	0.931–1.003	0.073	0.954	0.911–0.999	0.045
	Osteophyte score†† contralateral knee					
Knee extensors	0.978	0.956–1.000	0.048	0.972	0.946–0.999	0.042
Knee flexors	0.971	0.939–1.004	0.082	0.949	0.902–0.997	0.038

*Analysed with logistic regression, models with and without adjustment for sex and overweight are presented

†JSN grade refers to grade of joint space narrowing in the medial tibiofemoral compartment of the operated/contralateral knee; †† Osteophyte score refers to Osteophyte score of the medial tibiofemoral compartment of the knee

##### Osteophyte score in the medial compartment of the TF joint

**Fig. 4:**
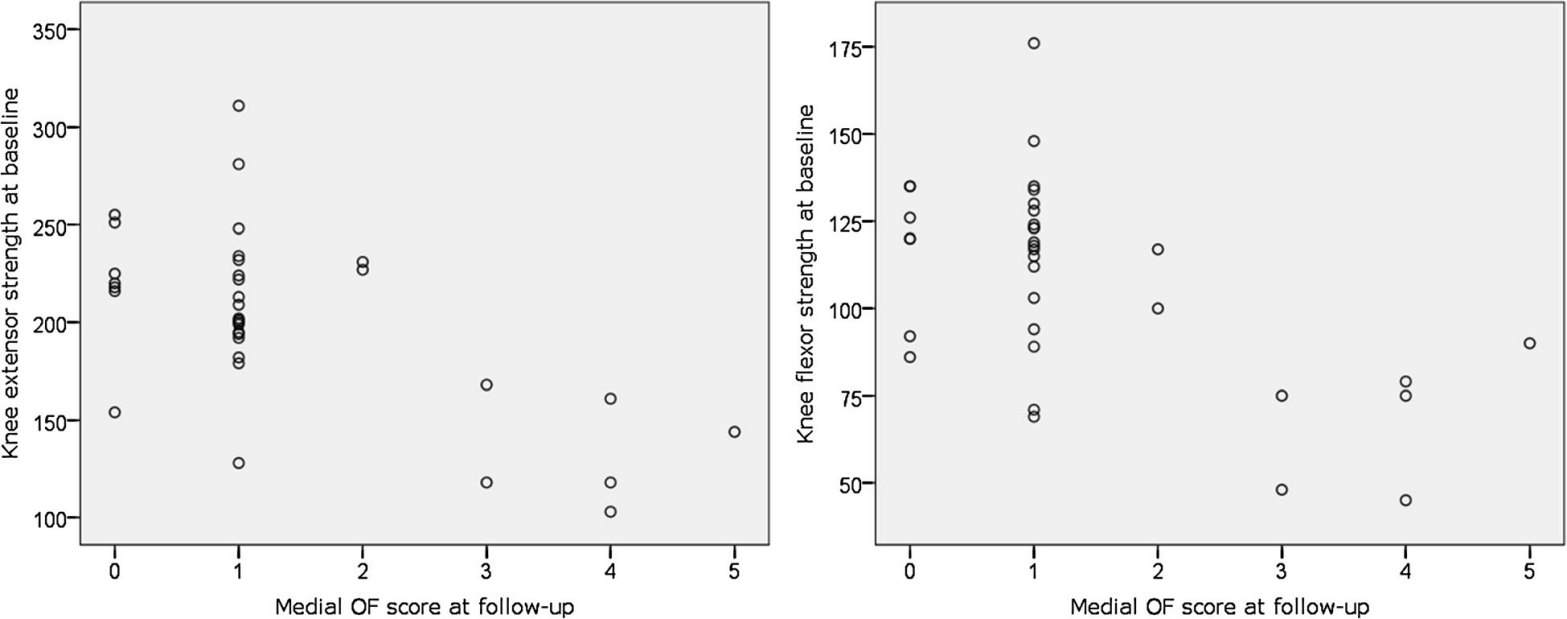
Relationship between baseline thigh muscle strength and osteophyte score in the operated knee at follow-up. *Medial OF score refers to Osteophyte score of the medial tibiofemoral compartment of the operated knee

Baseline knee extensor and knee flexor strength were negatively associated with osteophyte score in the operated knee at follow-up (Fig. 4), both in the unadjusted models, OR 0.966 and 0.936, *p* = 0.008 and 0.006, respectively (Table 2), and in the models with sex and overweight as control variables, OR 0.968 and 0. 931, *p* = 0.017 and 0.011, respectively (Table 2).

#### Contralateral knee

A negative association between baseline thigh muscle strength and radiographic OA features at follow-up was observed also in the contralateral knee. Figures for knee extensor strength and JSN grade: OR 0.961, p = 0.017 in the unadjusted model and OR 0.968, *p* = 0.027 in the adjusted model. The association between knee flexor strength and JSN grade was nonsignificant in the unadjusted model, OR 0.967, *p* = 0.073, but significant after adjustment for sex and overweight, OR 0.954, *p* = 0.045 (Table 2). Figures for knee extensor strength and osteophyte score: OR 0.978, *p* = 0.048 in the unadjusted model, and OR 0.972, *p* = 0.042 in the adjusted model. The association between knee flexor strength and osteophyte score was nonsignificant in the unadjusted model, OR 0.971, *p* = 0.082, but significant in the adjusted model, OR 0.949, *p* = 0.038 (Table 2).

#### Cross-sectional associations between muscle strength and radiographic OA features

Thigh muscle strength at follow-up was negatively associated to JSN grade and osteophyte score both in the operated and in the contralateral knee. Figures for knee extensor/knee flexor strength and JSN grade in the operated knee: OR 0.973 and 0.950 in the unadjusted models and OR 0.967 and 0.943 in the models adjusted for sex and overweight, *p* < 0.05. Corresponding figures for knee extensor /knee flexor strength and osteophyte score were OR 0.970 and 0.954 in the unadjusted models, OR 0.972 and 0.953 in the adjusted models, *p* < 0.05 (Table 3).

**Table 3:** Association between thigh muscle strength and radiographic knee OA features at follow-up*

	Unadjusted			Adjusted		
Predictor	OR	95% CI	*P*	OR	95% CI	*P*
Muscle strength, operated leg	JSN grade† operated knee					
Knee extensors	0.973	0.953–0.994	0.010	0.967	0.942–0.993	0.014
Knee flexors	0.950	0.914–0.988	0.009	0.943	0.899–0.989	0.016
	Osteophyte score†† operated knee					
Knee extensors	0.970	0.948–0.993	0.012	0.972	0.948–0.996	0.022
Knee flexors	0.954	0.916–0.993	0.022	0.953	0.913–0.994	0.025
Muscle strength, contralateral leg	JSN grade† contralateral knee					
Knee extensors	0.974	0.949–0.999	0.043	0.963	0.931–0.996	0.028
Knee flexors	0.950	0.903–0.998	0.043	0.940	0.886–0.998	0.043
	Osteophyte score†† contralateral knee					
Knee extensors	0.981	0.960–1.003	0.087	0.966	0.936–0.997	0.031
Knee flexors	0.956	0.914–1.000	0.049	0.939	0.885–0.997	0.038

*Analysed with logistic regression, models with and without adjustment for sex and overweight are presented.†JSN grade refers to grade of joint space narrowing in the medial tibiofemoral compartment of the operated/contralateral knee; ††Osteophyte score refers to osteophyte score of the medial tibiofemoral compartment of the knee

Figures for association between knee extensor strength/ knee flexor strength and JSN grade in the contralateral knee were OR 0.974 and 0.950 in the unadjusted models and OR 0.963 and 0.940 in the adjusted models, p < 0.05. The association between knee extensor strength and osteophyte score was nonsignificant in the unadjusted model, OR 0.981, *p* = 0.087, but significant in the adjusted model, OR 0.966, *p* = 0.031. Knee flexor strength was significantly associated with osteophyte score both in the unadjusted and adjusted model, OR 0.956 and 0.939, *p* < 0.05 (Table 3).

## Discussion

In the current study we wanted to examine if strong thigh muscles could protect the cartilage in subjects with high risk of later knee OA following meniscectomy. Our main finding was that lower thigh muscle strength four years after partial meniscectomy was associated with more severe radiographic OA changes in the medial tibiofemoral compartment of the operated and the contralateral knee 11 years later.

Weak but significant negative associations were generally seen between muscle strength and radiographic OA features in both operated and contralateral legs, longitudinally as well as cross-sectionally, suggesting a beneficial effect of strong thigh muscles on the knee joint in middle-aged subjects.

### Radiographic OA features

In this 15-year follow-up of middle-aged meniscectomized subjects, mean age at follow-up of 57 years, all had some radiographic OA features in the operated knee and two thirds of the subjects had OA features in the contralateral knee. As 80% of the subjects in this post meniscectomy cohort were classified as having radiographic OA in the operated knee at follow-up, we could not use presence or absence of OA as endpoint in the regression analyses. Instead we examined the association between thigh muscle strength at baseline and grade of separate radiographic OA features, JSN and osteophytes, using JSN grade and osteophyte score as dependent outcomes. The high frequency of structural changes could be explained by the selection of subjects who were middle-aged and had gone through surgery for a non-traumatic (degenerative) meniscal tear. As a degenerative meniscal tear has been suggested to be an early sign of knee OA [25], a high frequency of later radiographic changes could be expected in this cohort [26].

### Muscle weakness

Thigh muscles have been suggested to play an important part in the genesis of knee OA but few previous studies have focused on the relationship between muscle strength and knee OA after meniscectomy. Hall and co-workers, who used MRI to study the impact of knee joint loading and muscle strength on 2 year change in cartilage integrity after APM found no significant association for quadriceps strength [27]. Hall’s findings at 2 years after surgery partly contravene ours, but the MOST study examining (unoperated) subjects with meniscal pathology found that lower knee extensor strength was associated with a higher risk of radiographic knee OA in women but not in men 7 years later [28]. Interestingly, Øiestad and co-workers, who studied the role of muscle strength after anterior cruciate ligament (ACL) reconstruction, found no association between knee extensor weakness after surgery and radiographic knee OA 10–15 years later [29]. It may be that good thigh muscles, that are able to protect the cartilage by attenuating impact loads, may be more important after meniscectomy in the middle aged than after ACL reconstruction in young adults who generally are more fit. In general, muscle strength levels start to decrease in the fifth decade of life [30] resulting in less spare muscle capacity, which may be more obvious after a joint injury. The important role of the meniscus in OA development is supported by Neuman et al. who reported that isolated ACL-injury was associated with lower incidence of radiographic knee OA than combined ACL- and meniscus injury at 10–15 years after ACL injury [31].

After meniscectomy the cartilage may be unable to cope with normal joint loading, especially in the absence of well-functioning thigh muscles that are capable of controlling the forces acting on the joint [16]. This study focused on muscle strength, but factors that we did not assess, like knee joint loading and muscle activation patterns, might have played a role, as minor changes in kinematics, kinetics, and neuromuscular activity have been reported after meniscectomy [18, 32]. In patients with knee OA quadriceps muscle weakness previously has been attributed to disuse atrophy as a consequence of joint pain, but as quadriceps weakness has been found in asymptomatic subjects with radiographic knee OA other causal factors like muscle dysfunction and impaired proprioception have been proposed [33, 34].

### Contralateral knee

Radiographic OA features were more prevalent in operated knee joints than in contralateral knees. Structural changes that were classified as radiographic TF OA were about twice as frequent in the operated knee as compared to the contralateral knee, which is in agreement with the findings from a recent review [8]. As all subjects were treated for a non-traumatic (degenerative) type of meniscus tear, previously shown to be associated with hand OA [35], it is possible that genetic factors predisposing for OA played a role.

Our hypothesis was that the meniscectomized knee would be more dependent of adequate muscle strength than the contralateral knee, but we found similar longitudinal and cross-sectional negative associations between thigh muscle strength and OA features for the contralateral knee as for the operated. This may be interpreted as that good muscle strength is important for the cartilage in injured as well as uninjured knees in subjects with increased risk of OA.

### Limitations

This study has important limitations. First, the study sample was small and 25% were lost to follow-up at 11 years. The original cohort consisted of only 45 patients and 34 of them (75%) participated in the follow-up. However, the eleven patients that did not participate had similar gender distribution, age, BMI, and baseline muscle strength as the rest of the group. Second, we performed no radiographic examination of the knee joint at the baseline examination, which precludes longitudinal comparisons. However, since the arthroscopy reports gave no evidence of severe cartilage changes, we find it unlikely that a substantial portion of the patients had developed radiographic OA already at the time of baseline examinations. Still, we cannot completely rule out the possibility of reverse causality, i.e. that pain and early (pre-radiographic) OA may have led to low muscle strength already at the baseline exam.

## Conclusion

Better thigh muscle strength 4 years after medial meniscectomy was associated with less severe radiographic OA changes in the medial tibiofemoral compartment of both the operated and the contralateral knee 11 years later. Our findings may suggest that strong thigh muscles can help to preserve joint integrity in middle-aged subjects at risk of knee OA.

## Data Availability

The datasets used and/or analysed during the current study are available from the corresponding author on reasonable request.

## Abbreviations

ACL: Anterior cruciate ligament
APM: Arthroscopic partial meniscectomy
dGEMRIC: delayed Gadolineum-Enhanced MRI of Cartilage
JSN: Joint space narrowing
OA: Osteoarthritis
OF: osteophytes
PT: peak torque
TF: tibiofemoral
